# Detection of meteorological influence on bread wheat quality in Hebei province, China based on the gradient boosting decision tree

**DOI:** 10.3389/fpls.2023.1083665

**Published:** 2023-05-30

**Authors:** Xinyue Zhang, Keyao Chen, Kuo Li

**Affiliations:** ^1^ Institute of Environment and Sustainable Development in Agriculture, Chinese Academy of Agricultural Sciences, Beijing, China; ^2^ National Climate Center, China Meteorological Administration, Beijing, China

**Keywords:** grain protein content, winter wheat, meteorological influence, Hebei province, gradient boosting decision tree

## Abstract

Wheat grain quality is equivalent to grain yield in terms of ensuring food security under climate change but has received less attention. Identifying critical meteorological conditions in key phenological periods to account for the variability in grain protein content (GPC) can provide insight into linkages between climate change and wheat quality. The wheat GPC data from different counties of Hebei Province, China during 2006-2018 and corresponding observational meteorological data were used in our study. Through a fitted gradient boosting decision tree model, latitude of the study area, accumulated sunlight hours during the growth season, accumulated temperature and averaged relative humidity from filling to maturity were suggested as the most relevant influencing variables. The relationship between GPC and latitude was distinguished between areas north and south of 38.0° N. GPC decreased with the increasing latitude in areas south of 38.0° N, where at least accumulated temperatures of 515°C from filling to maturity were preferred to maintain high GPC. Besides, averaged relative humidity during the same phenological period exceeding 59% could generate an extra benefit to GPC here. However, GPC increased with increasing latitude in areas north of 38.0° N and was mainly attributed to more than 1500 sunlight hours during the growth season. Our findings that different meteorological factors played a major role in deciding regional wheat quality provided a scientific basis for adopting better regional planning and developing adaptive strategies to minimize climate impacts.

## 1 Introduction

Wheat (*Triticum aestivum* L.) is the second most important staple crop in China, with an annual grain production of more than 134 million tons (FAOSTAT, 2017). Because agricultural production is very susceptible to climate change, a growing body of research has revealed the impact of meteorological factors on wheat grain yield from local to regional scales (Dong et al., 2017; Kamir et al., 2020; Zahra et al., 2023). In a changing climate, delivering grain with consistent quality to ensure a stable nutrition supply has already been threatened considerably (Nuttall et al., 2017; Santos et al., 2023). However, less attention has been given to the impact of meteorological factors on grain quality, even though they are comparable to grain yield from a food security perspective (Haddad et al., 2016). As the primary quality index in the grain of cereal crops, protein content is directly related to the plant-based protein needed by human beings.

Mature wheat grain usually contains 8% to 15% protein content, a small fraction but a significant determinant of end-use quality (Békés and Wrigley, 2016). For example, GPC lower than 13% belongs to a weak gluten wheat cultivar which is mainly for making cakes and biscuits. GPC between 13% and 14% is classified as a medium gluten cultivar, which fundamentally meets the needs of making noodles and bread, while GPC higher than 14% belongs to strong gluten wheat cultivar which can produce al dente noodles and bread. Although GPC basically depends on the wheat cultivar (e.g. whether it belongs to a weak, medium, or strong gluten cultivar), it is also greatly modified by the living environment, especially meteorological conditions (Asseng et al., 2019). Previous studies have observed changes in wheat GPC at different experimental sites caused by several meteorological factors (Lee et al., 2013; Toscano et al., 2015). For example, it is common for wheat to experience high temperature stress during grain filling periods around the world (Zhang et al., 2019). A great number of studies suggested that moderate high-temperature stress leads to an increase in GPC (Barutcular et al., 2016; Zhang et al., 2019). However, other studies observed no effect or even a negative effect of high temperature on GPC during the same phenological periods (Fernie et al., 2022). The effect of high temperature on wheat quality depends not only on the differences in heat tolerance among wheat cultivars but also to a large extent on the specific temperature conditions that wheat encounters (Wrigley, 2006; Zhang et al., 2019; Fernie et al., 2022). Although temperatures strongly influence GPC, other studies found that GPC is best explained by variability in light intensity and relative humidity (Li et al., 2005). The mechanisms that drive this variation across experiments remain poorly understood, creating uncertainty in climate projections and making extrapolation of these results to a regional scale problematic. Moreover, few studies have compared the relative importance of multiple meteorological variables during different wheat phenological periods. A great majority of previous work focused on studying meteorological conditions during the grain filling period mainly. Meteorological conditions prior to grain filling were also related to GPC, but their effects were often underestimated. At least half of the nitrogen in grain comes from the redistribution of nitrogen present in different vegetative organs before anthesis (Zhang et al., 1997). Since the formation of protein in wheat grain is influenced by crop growth, nitrogen uptake and redistribution processes during critical growth periods, meteorological conditions that influence these processes will also play a vital role in determining grain protein.

As the major wheat production region, the North China Plain produced more than 70% of the total wheat production in China (Zhang and Lu, 2019). Winter wheat planted in this area is all medium to strong gluten cultivars, thus generating higher protein content in grains compared with other winter wheat planting areas in China (Ma et al., 2021; Zhou et al., 2021). Hebei Province is one of the core regions of wheat production in North China Plain, and also the key planting area for high GPC wheat. The local government attached great importance to the matter of developing a strong gluten wheat industry and released a series of relevant policy documents, such as the latest Promotion Plan for Quality and Efficiency Improvement of Strong Gluten Wheat Industry in Hebei Province (2019-2022). Nowadays, the most prominent problem for medium to strong gluten wheat in China is that GPC is unstable and sometimes considerably lower than expected (Ma et al., 2021). This problem may further be amplified by climate change. With a large population and limited arable land, agriculture is quite vulnerable to climate change in China. The adverse effects of climate change include frequent floods and droughts caused by uneven spatial and temporal distribution of water resources, more severe high temperature damage, intensified outbreaks of pests and diseases, and more climate extreme events (China’s Policies and Actions for Addressing Climate Change, 2019). The past five decades (1956-2007) have witnessed an upward trend in mean annual temperature of Hebei Province. Besides, annual precipitation generally showed a decreasing trend, especially in the summer season (Liu et al., 2014). Identifying critical meteorological conditions in key phenological periods to account for GPC variation can provide insight into effects of climate change on wheat quality in Hebei Province, as well as develop coping strategies to minimize impacts of future warming and drying trends for this region.

In an attempt to address the issues mentioned before, county-level wheat quality data in Hebei Province was extracted from the China Wheat Quality Report 2006~2018 released by the Ministry of Agriculture and Rural Affairs of China. Corresponding ground-based observation of daily meteorological data was obtained from China Meteorological Data Service Center, National Meteorological Information Center. Our main objective is to evaluate the relative importance of meteorological parameters during different phenological periods and find the notable determinants of GPC in Hebei Province. A recent study based on the same wheat data on this topic suggested that the effect of air temperature, precipitation, and solar radiation on wheat GPC is very limited in the North China Plain, accounting for no more than 8% of variability in GPC (Zhou et al., 2021). This study differs from Zhou et al. (2021)’s work by using observed meteorological data instead of gridded meteorological data and focusing on Hebei Province located within the North China Plain. The resolution of gridded meteorological data was much coarser than ground based observations, which may be incapable of exploring the driving factors of GPC at a smaller regional scale. Furthermore, the accumulation of temperature or solar radiation during different phenological periods are important for crops to complete relevant growth stages, but they are not taken into consideration in previous work. In our study, we included five types of meteorological parameters, namely, mean air temperature, accumulated air temperatures, accumulated precipitation, mean relative air humidity and accumulated sunlight hours during different key phenological periods. These parameters could increase the understanding of GPC variation in response to different meteorological factors.

## 2 Materials and methods

### 2.1 Study area and data collection

**Figure 1:**
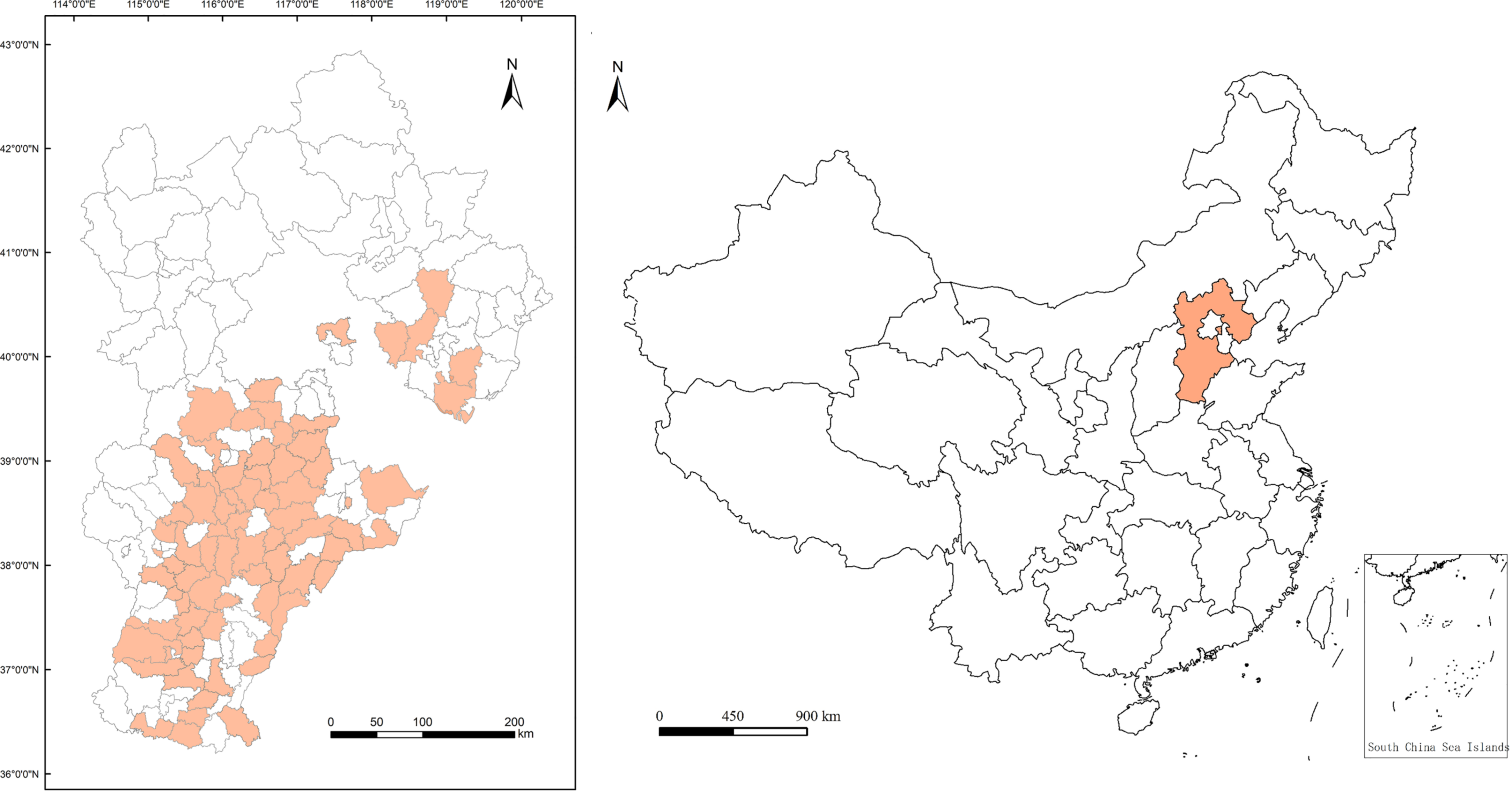
Two-dimensional partial dependence plot (2D PDP) for revealing the interaction effect of latitude and relative humidity on grain protein content (GPC). The top horizontal label represents the variation in latitude (Lat). The left vertical label represents the variation in relative humidity (Havg) from filling to maturity. The right vertical label represents the variation in GPC.

Hebei Province (36°01′ ~ 42°37′ N, 113°04′ ~ 119°53′ E) is located in the North China Plain (**Figure 1**), with a total land area of 188,800 square kilometers. It is the main bread wheat production region for medium to strong gluten winter wheat and important commodity grain base of China. This region has a typical temperate continental monsoon climate with four distinct seasons. The annual mean temperature (MAT) and annual precipitation (MAP) during recent decade range from -0.3°C to 14°C and 350 mm to 770 mm, respectively.

We obtained county-level bread wheat data from publicly available reports of the China Wheat Quality Report 2006~2018 released by the Ministry of Agriculture and Rural Affairs of the People’s Republic China (www.moa.gov.cn), including winter wheat cultivar, county name where the wheat was sown, harvest year, and GPC measured by the semimicro Kjeldahl method according to China Agricultural Industry Standard NY/T 3–1982. In total, 306 winter wheat samples from 65 counties in Hebei were collected and used in our analyses. The geographic distribution (e.g., latitude and longitude) of each county is shown in **Figure 1**. Meteorological data were downloaded from the platform of the China Meteorological Data Service Center, National Meteorological Information Centre (http://data.cma.cn). They were ground-based observation data at county level, including daily mean, maximum and minimum air temperature, daily relative air humidity, daily precipitation, and daily sunlight hours.

### 2.2 Study method

To address the scientific question on how meteorological conditions in critical phenological periods affect GPC, identifying growth stages of winter wheat is the first step. After knowing those stages, we can obtain their meteorological conditions. Due to absence of relevant records (e.g., sowing, regreening, jointing, flowering, filling and maturity dates) in China Wheat Quality Report, we acquired various growth stages for each winter-wheat sample based on previous findings (Yan et al., 2000; Li et al., 2012; Yue et al., 2012; Kang et al., 2015; Shan et al., 2020).

The sowing date of winter wheat in the Hebei Province varied from late September to mid-October. To determine the specific sowing date, we considered the growing degree days (GDD) required by winter wheat to complete physiological development before winter dormancy (Li et al., 2012). GDD is a thermal time concept, reflecting the energy requirement for crop growth to reach maturity (Tao et al., 2012). It is calculated as summation of daily mean air temperature minus baseline temperature for crop growth (0°C for winter wheat) during a determined period of time. If a daily mean air temperature is below 0°C, the GDD value for that day is recorded as zero. The suitable GDD of winter wheat from sowing to winter dormancy is 570 ~ 770 GDD (Li et al., 2012). The sowing date was presumed as the ideal sowing date for winter wheat to live through winter safely. We need sowing dates only because we want to calculate averaged values of meteorological variables from sowing to maturity (about 250 days) in our analysis. The averaged results were fairly insensitive to the choice of sowing date varied from late September to mid-October. Moreover, the rest meteorological variables we used for analysis weren’t related to the sowing dates and the acquiring of other phenological dates was also independent of the sowing dates.

Regression models constructed by Shan et al. (2020) and Kang et al. (2015) were applied to determine regreening and maturity dates, respectively. The former regression model was constructed by using observation data from 11 agrometeorological stations in Hebei Province from 1981-2018 to forecast the regreening date of winter wheat (Shan et al., 2020). Their results show that the key factor to determinate the regreening date is the negative accumulation temperatures during the winter dormancy periods. The regression equation is:


, (1) Y= −0.0703X+20.339

where, X is the negative accumulation temperatures, calculated from the summation of daily mean air temperature when it is below 0°C from 1 October to 15 February of the following year, and Y is the regreening date in numeric form (Shan et al., 2020). For example, Y=1 means 1 February, Y=2 means 2 February, and the rest can be deduced by analogy.

The latter model also focuses on the winter wheat in Hebei Province, simulating maturity based on the relationship between growth rate and GDD (Kang et al., 2015). The regression equation is:


, (2) $$Growth\;rate=0.0014+0.0006GDD_{regreening\;to\;maturity}$$

where, $GDD_{regreening\;to\;maturity}$ is the summation of daily mean air temperature when it is above 0°C from regreening to maturity. The regreening date is obtained from field observation in Kang et al. (2015)’s study but generated from equation (1) in our study. After that, the maturity date is determined by this equation till Growth rate reaches 1.

The generation of both jointing and filling dates also depends on the regreening date, since long term observations suggest that it was approximately 380 and 1160 GDD from regreening to jointing and grain filling, respectively (Yue et al., 2012). Once the filling date was fixed, the flowering date could be calculated in reverse since at least 300 GDD was required from flowering to filling (Yan et al., 2000).

Daily meteorological data were processed according to different phenological periods in order to relate seasonal climate conditions to yearly wheat-quality. Finally, 29 variables were established as potential influencing factors of GPC. Two variables were longitude and latitude, providing geographic location for the studied winter-wheat counties. The remaining variables were meteorological factors during different growing periods. Meteorological conditions in two periods were not taken into consideration in our study. One is the winter dormancy period when winter wheat ceases to grow. Another is the period from regreening to jointing when the averaged meteorological conditions during this period had smaller variation compared to other periods. Pre-analysis results showed its effect on GPC is hard to be detected and too many variable inputs would reduce the model performance. Therefore, we excluded the meteorological data during regreening to jointing period. For wheat samples of different cultivars but collected from the same county in the same year, there would be the same geographic information and meteorological conditions for different GPC. Thus, we used the averaged values of GPC to reduce model fitting uncertainty. The sample size consequently shirked from the original 306 to 176.

Rapid development of machine learning in recent years provides a new technique for addressing regression problems and detecting general patterns. Integrated learning is one the most widely adopted supervised machine learning algorithms. We employed the gradient boosting decision tree (GBDT) model, a typical integrated learning method, in this study because of its excellent regression ability with smaller data size (Zhang and Haghani, 2015). In addition, another important reason we adopted GBDT instead of regression analyses such as ordinary least squares (OSL) is that the output of GBDT is not affected by the presence of correlated variables. The mathematic function of GBDT model can be expressed as below:


, (3) $$f_{k}\left(x\right)=\sum_{k=1}^{N}T\left(x;\theta_{k}\right)$$

where, $T\left(x;\theta_{k}\right)$ is the classification and regression tree (CART) algorithm, $\theta_{k}\;$ and N represent the parameter and number of CART. $f_{k}\left(x\right)$ is the predicted value of *k-th* CART. Function (3) can also be expressed in a recursive form by introducing forward step algorithm:


, (4) $$f_{k}\left(x\right)=f_{k-1}\left(x\right)+T\left(x;\theta_{k}\right)$$

Assuming that $\;y_{i}\;$ is the observed value of sample  i , then $f_{k}\left(x_{i}\right)\;$ is the predicted value of sample i . The loss function L , used for estimating the difference between the observed and predicted value by calculating the residual sum of squares, can be expressed as:


, (5) $$J=\sum_{i=1}^{N}L\left(y_{i},\;f_{k}\left(x_{i}\right)\right)=\sum_{i=1}^{N}\frac{1}{2}{\left(y_{i}-f_{k}\left(x_{i}\right)\right)}^{2}$$

When training the *k-th* CART, the following loss function need to be minimized as much as possible:


, (6) $$J=\sum_{i=1}^{N}L\left(\;y_{i},f_{k-1}\left(x_{i}\right)+T\left(x;\theta_{k}\right)\right)$$

The method of gradient descent can be used to reduce the objective loss function (6). The gradient of the objective function to $\;f_{k-1}$ is denoted as following:


, (7) $$\frac{\partial J}{\partial f_{k-1}}$$

Then the parameter optimization form can be expressed as following:


, (8) $$f_{k}\left(x_{i}\right)=f_{k-1}\left(x_{i}\right)-alpha\cdot \frac{\partial J}{\partial f_{k-1}}$$

where, alpha  is the learning rate.

According to function (4) and (8), it denoted that


, (9) $$T\left(x;\theta_{k}\right)=\;-alpha\cdot \frac{\partial J}{\partial f_{k-1}}$$

Where function (9) indicates the *k-th* CART is learned for the negative gradient direction of the objective function.

According to function (5) and (9), it denoted that:


, (10) $$T\left(x;\theta_{k}\right)=\;-alpha\cdot \frac{\partial J}{\partial f_{k-1}}=\;y_{i}-alpha\cdot f_{k-1}\left(x_{i}\right)$$

where, function (10) indicates that each CART under the GBDT model is the residual of fitting the previous round of training results.

Then the parameters of the CART can be obtained by minimizing the loss function:


, (11) $${\hat{\theta }}_{k}=argmin\sum_{i=1}^{N}L\left(\;y_{i},f_{k-1}\left(x_{i}\right)+T\left(x;\theta_{k}\right)\right)$$

Through multiple iterations, training results of the CART are continuously updated to generate the optimal prediction of the model.

The feature importance of feature $x_{j}$ in the overall model is calculated as:


, (12) $$J_{j}^{2}=\frac{1}{M}\sum_{m=1}^{M}J_{j}^{2}\left(T_{m}\right)$$

where, M is the number of decision trees for the model, T represents one decision tree.

The feature importance of feature $x_{j}$ on a separate tree is calculated as:


, (4) $$J_{j}^{2}\left(T\right)=\sum_{t=1}^{L-1}I_{t}^{2}loss\left(v_{t}=j\right)$$

where, L−1 is the number of non-leaf node in the decision tree; $v_{t}$ is the selected character when the internal node t splits; $I_{t}^{2}$ is the reduced amount of loss function when the internal node t splits; loss is the loss function.

To perform the GBDT model, all samples were randomly split into 80% training data and 20% testing data. The constructed GBDT model can be used to predict the variability in regional GPC and identify the most important variables affecting GPC based on Gini importance. The Gini importance of each variable is computed as the normalized total reduction of the criterion brought by that variable to reflect variable relevance (the higher Gini, the more relevant). After the model was fitted, partial dependence plots (PDP) were drawn for identified important variables to visualize how a variable affected predictions, solving the problem that Gini importance can only show what variables affect predictions the most. PDP can separate the effect of individual variables. To be more specific, a selected variable is altered repeatedly while other variables are kept unchanged for each row of the dataset to make a series of predictions based on the fitted GBDT model. The altered times and specific values for the selected variable are automatically produced and shown in the x axis of the PDP. The average predictions for multiple rows are plotted on the vertical axis. Therefore, the y axis of the PDP is interpreted as the change in model prediction compared with the baseline or leftmost value. The resulted points are labeled as “Number of unique grid points” in PDP. Considering interactions between variables, two-dimensional PDP (2D PDP) are further created to visualize the combined effect of two variables on the predictions at the same time.

The GBDT model, PDP and 2D PDP were implemented by Python v3.6 (Python Software Foundation, 2020). All statistical analyses were completed in R 3.6.2 (R software, 2019). The figure showing the distribution of sampling counties was drawn by ArcGIS 10.0 (Esri Inc., California, USA). Other figures were drawn by R 3.6.2 with the ggplot2 package.

## 3 Results

### 3.1 Variation of GPC

Winter wheat GPC varied from 11.7% to 17.8% depending on years and locations. Considering the Chinese National Standard GB/T 17320-1998 for GPC: high quality with GPC values higher than 14%, medium quality between 13% and 14% and low quality below 13%. The GPC for approximately 68% of the total samples reached high quality, with a mean value of 15.1% ± 0.9%. Approximately 24% of the total samples had medium quality, with a mean GPC value of 13.6% ± 0.3%. The remaining 24 samples were classified to low quality. There was significant interannual variation in GPC (*p*< 0.001), with the lowest value found in 2011 (13.5% ± 0.7%) and the highest value found in 2018 (15.0% ± 1.1%). There were a higher number of winter wheat samples in last five years of the reporting period, corresponding to an increased priority in local government to collect this information.

### 3.2 Variation of meteorological conditions

For the growing period from sowing to maturity, the mean, maximum and minimum temperature, relative humidity, precipitation and sunlight hours were 8.7 ± 0.6°C, 14.8 ± 0.7°C and 3.6 ± 0.9°C,57.8% ± 4.7% 158.2 ± 64.2 mm and 1562.7 ± 201.4 h, respectively. Temperature, relative humidity and sunlight hours did not show significant interannual variability Precipitation fluctuated significantly among sampling years, mainly due to the highest amount of rainfall in 2018. A heavy precipitation event occurred on 21 and 22 April 2018. Total rainfall amount in that event was 1.2 times above normal. Except for that extreme event of precipitation in 2018, precipitation varies within the normal range during the study period. Detailed information on meteorological variables during different wheat phenological periods is shown in **Table 1**.

**Table 1:** Basic information on meteorological variables during different wheat phenological periods.

Phenological period	Temperature (°C)	Relative humidity (%)	Precipitation (mm)	Sunlight hours (h)
Regreening to maturity	16.8 ± 0.5 (3.2%)	54.2 ± 5.4 (9.9%)	100.9 ± 49.5 (49.1%)	781.1 ± 94.0 (12.0%)
Jointing to flowering	17.9 ± 0.9 (4.8%)	56.4 ± 7.8 (13.8%)	43.0 ± 35.6 (82.7%)	244.9 ± 32.3 (13.2%)
Flowering to filling	21.6 ± 1.5 (7.1%)	58.8 ± 11.5 (19.6%)	13.3 ± 17.5 (131.7%)	82.8 ± 25.0 (30.2%)
Filling to maturity	24.4 ± 0.9 (3.7%)	55.9 ± 5.6 (10.0%)	31.3 ± 21.6 (69.0%)	187.6 ± 28.5 (15.2%)

The results are presented as the mean value ± standard deviation (coefficient of variation).

### 3.3 Model performance

**Figure 2:**
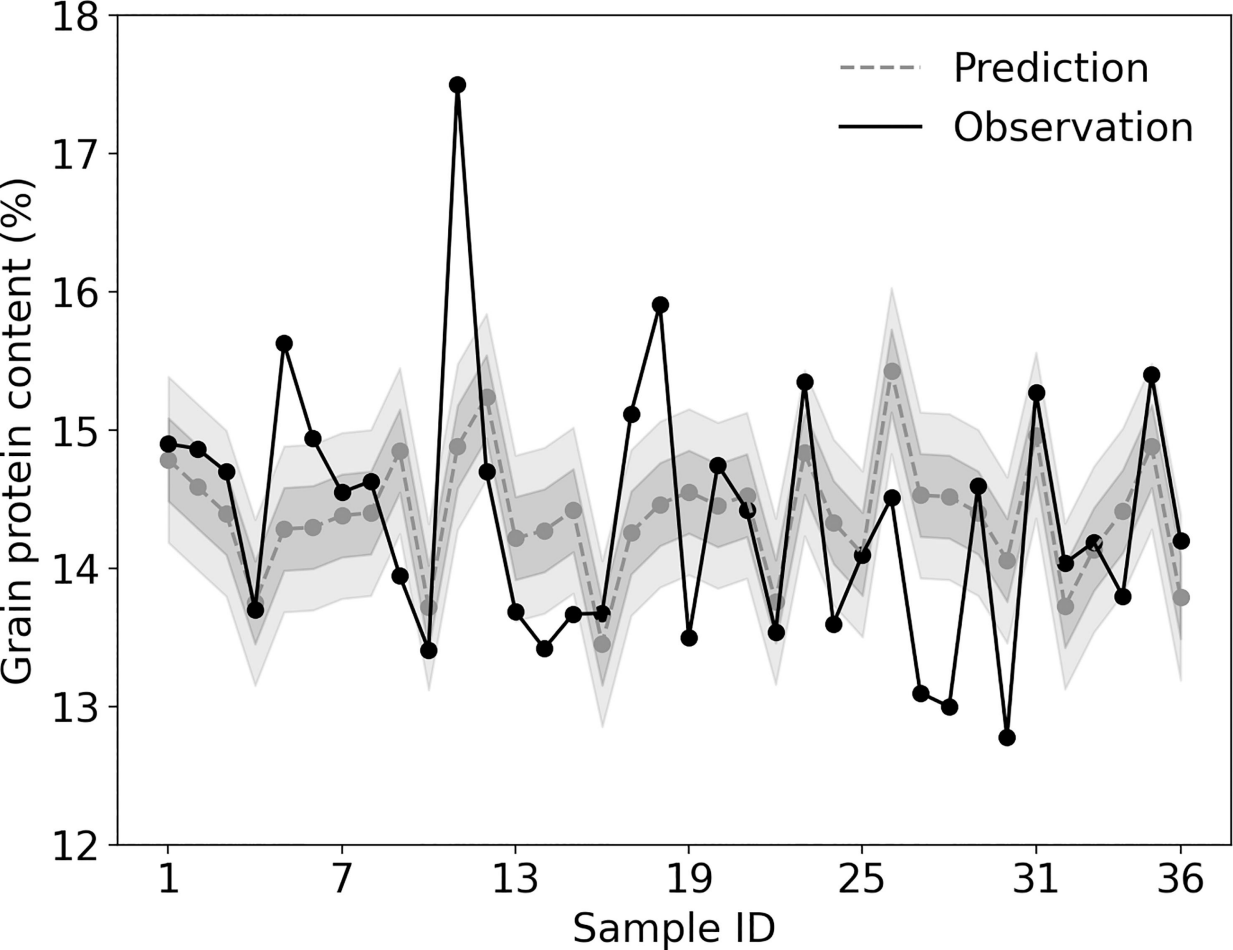
Distribution of sampled counties in Hebei Province (left) and the geographic location of Hebei Province in China (right).

We used the test dataset to evaluate GBDT model performance. The mean value of model predicted GPC in the test dataset was 14.4% ± 0.4%, close to the observed GPC (14.4% ± 0.9%). The predicted extent however was smaller than the observed. The predicted GPC varied from 13.5% to 15.4%, whereas the observed GPC varied from 12.8% to 17.5%. GBDT model performance was visualized in **Figure 2** Several commonly used model performance metrics were calculated. The coefficient of determination (R^2^) was 19.7%. The mean absolute error was 0.63%, which represented the mean value of absolute error between the predicted value and the observed value in the test dataset. If the maximum permissible error was set as 0.6% (similar to the mean absolute error), the accuracy rate was approximately 58.3%.

### 3.4 Key influencing factors of GPC

Although the GBDT model was not accurate enough to predict GPC precisely, it was fairly efficient in identifying the factors that are statistically significant in affecting the variability of GPC. We listed the first nine out of 29 variables that together weighted 75% of the total Gini importance scoring (**Table 2**). Latitude was the most important factor to decide GPC (**Table 2**). Apart from latitude, sunlight hours were the most important meteorological factor to influence GPC. Sunlight hours ranked second and appeared three times in **Table 2**. GDD ranked third and appeared twice in **Table 2**. Averaged temperature of the growth stage from jointing to flowering also exerted some effect. Relative humidity and precipitation, two meteorological variables related to water conditions, both show important effects on GPC from the filling to maturity stages. Four out of the nine indicators that have an important effect on GPC were from the specific growth stage of filling to maturity.

**Table 2:** The Gini importance for the first nine variables identified by the gradient boosting decision tree (GBDT) model.

Rank	Variable name	Phenological period	Gini importance
**1**	**Latitude**	**/**	**0.147**
**2**	**Sunlight hours**	**Sowing to maturity**	**0.143**
**3**	**Growing degree days**	**Filling to maturity**	**0.118**
**4**	**Relative humidity**	**Filling to maturity**	**0.094**
5	Temperature	Jointing to flowering	0.062
6	Growing degree days	Sowing to maturity	0.050
7	Sunlight hours	Filling to maturity	0.050
8	Sunlight hours	Jointing to flowering	0.040
9	Precipitation	Filling to maturity	0.040

The symbol “/” means no involvement of phenological period. The most important variables were highlighted in bold.

**Figure 3:**
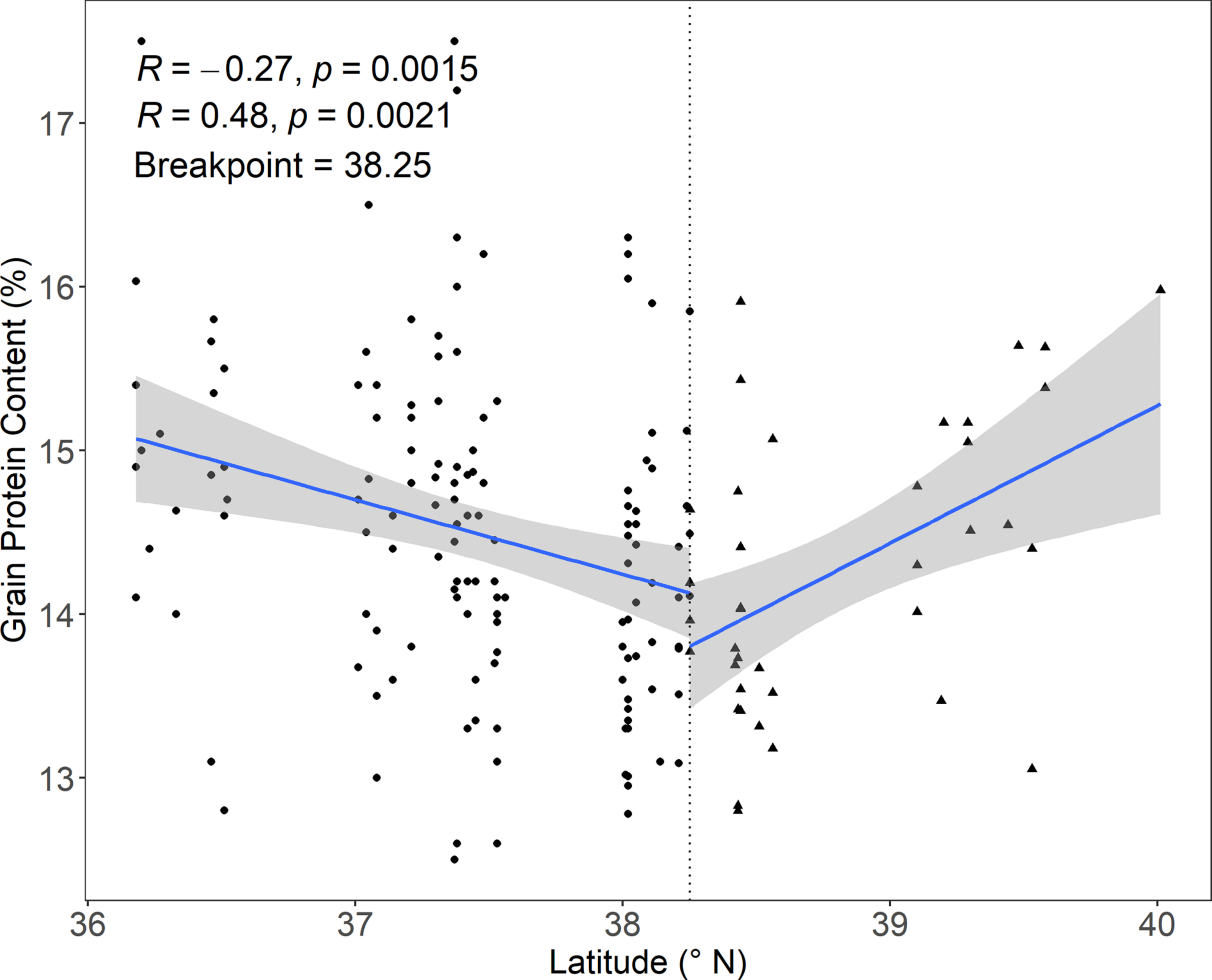
Performance of the gradient boosting decision tree (GBDT) model in predicting grain protein content (GPC) for each sample of the test dataset. The solid line shows the observed value from the test dataset. The dashed line and shade represent the model predicted value ± maximum permissible error. The inside shade (dark shade) means that the maximum permissible error is 0.3%, whereas the outside shade (light shade) means that it is 0.6%.

**Figure 4:**
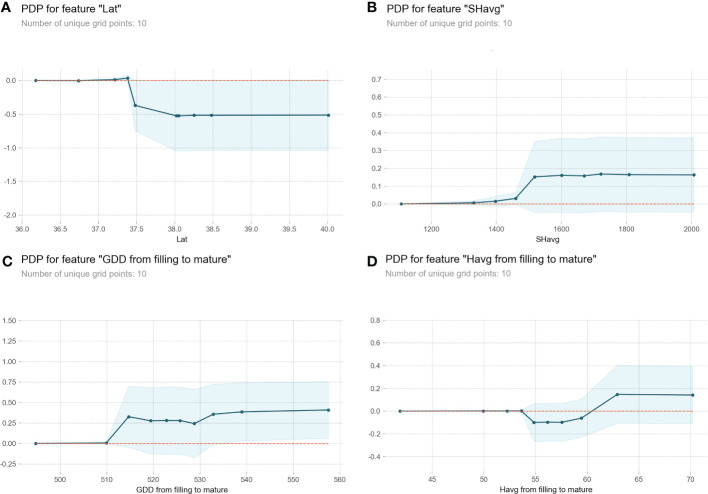
Partial dependency plots (PDPs) for the four most influential variables identified from the GBDT model. The y-axis of each plot represents the change in model prediction compared with the baseline value. The x-axis represents different variables: **(A)** Latitude (Lat); **(B)** Sunlight hours (SHavg) from sowing to maturity; **(C)** Growing degree days (GDD) from filling to maturity; **(D)** Relative humidity (Havg) from filling to maturity. The light blue shade represents the 95% confidence interval.

A partial dependency plot (PDP) was employed to explore the relationship of the top four most influential variables for GPC (**Figure 3**). We identified the top four variables as the most relevant factors to account for the variability of GPC because they comprised half of the total Gini importance scoring (**Table 2**). According to the y-axis of PDP, the top four variables exerted different influences on GPC. There was a clear decreasing trend of GPC with increasing latitude until it reached approximately 38.0° N (**Figure 3A**). When sunlight hours of the whole growth stage accumulated to 1500 h, GPC was significantly improved (**Figure 3B**). GDD from filling to maturity promoted GPC once it reached above 515 GDD. There was a slight decreasing trend of GPC with higher GDD from 515 to 530 GDD (**Figure 4C**). Relative humidity from filling to maturity was the only meteorological variable that generated a different effect on GPC. The range of relative humidity during filling to maturity stage was 42% to 70%. **Figure 3D** clearly showed that GPC was unaffected when relative humidity increased from 42% to 55%. A turning point appeared at around 55%, which indicated an obvious decrease in GPC. And the GPC remained at the low value until the relative humidity increased to 58%. GPC increased with the increase of relative humidity from 58% to 63% and kept the high value when relative humidity increased from 63% to 70%.

**Figure 5:**
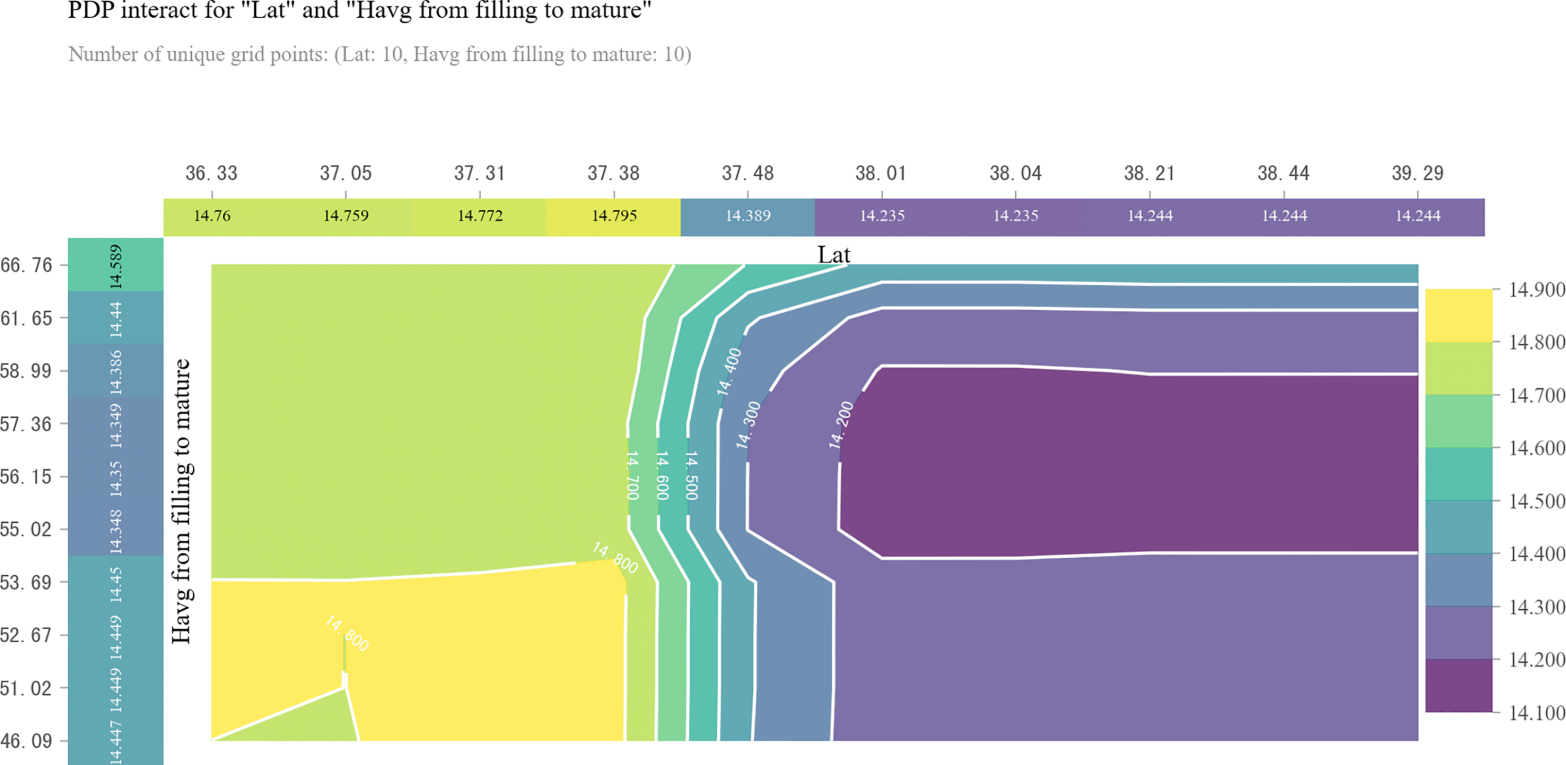
Two-dimensional Partial dependence plot (2D PDP) for revealing the interaction effect of latitude and growing degree days (GDD) on grain protein content (GPC). The top horizontal label represents the variation in latitude (Lat). The left vertical label represents the variation in GDD from filling to maturity. The right vertical label represents the variation in GPC.

**Figure 6:**
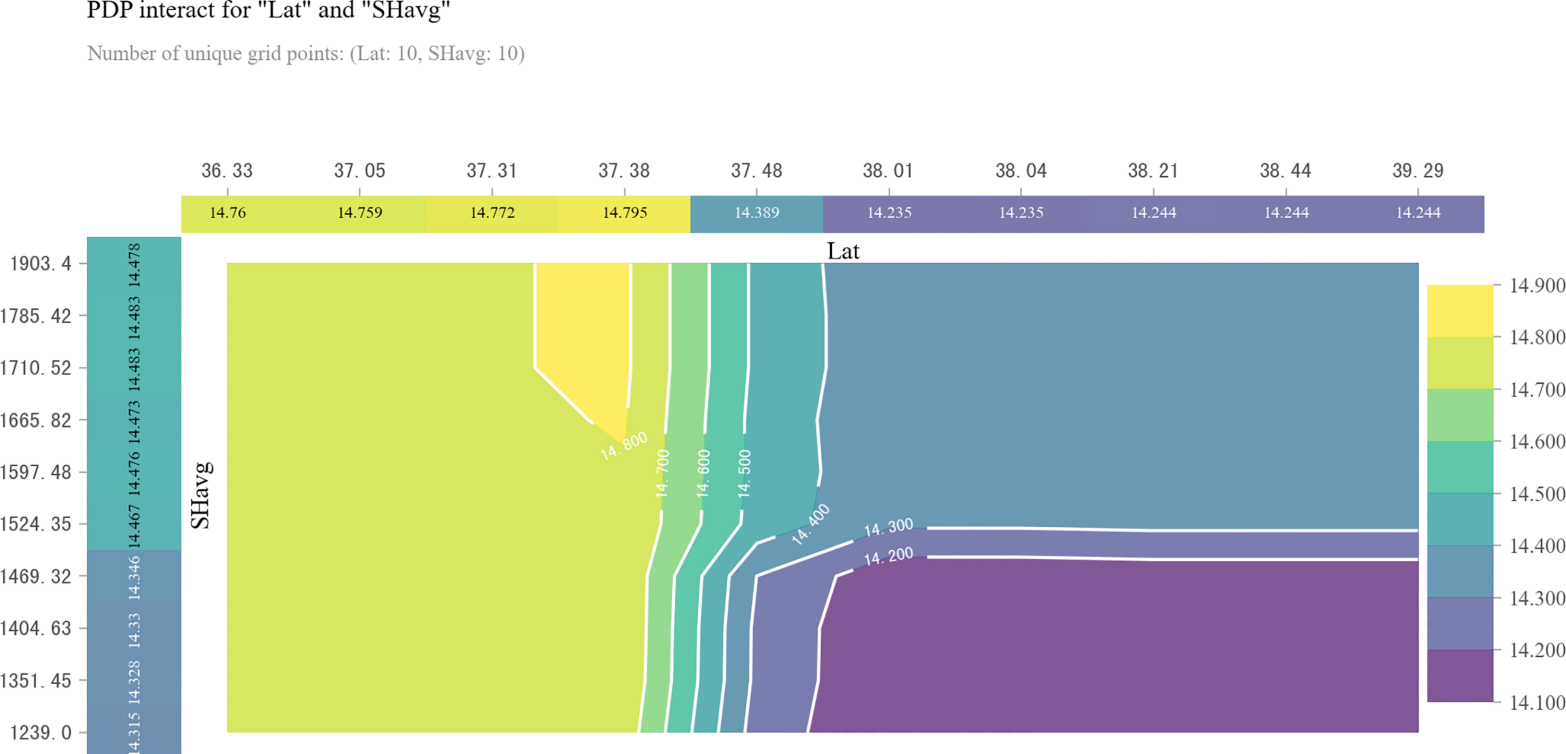
The piecewise linear regression between latitude and GPC. The blue lines represent the fitted linear regression line. The gray area around each blue line represents the 95% confidence interval.

To further test the threshold of above four most important variables, we performed piecewise linear regressions advocated by Toms and Lesperance (2003). There was a breakpoint of latitude found by the piecewise regression successfully, which was 38.3° N, close to the threshold identified by the GBDT model (**Figure 5**). Then, we investigated the interaction between latitude and the other three most relevant meteorological parameters on GPC by 2D PDP. GPC in areas south of 38.0° N increased with increasing latitude regardless of changes in sunlight hours (**Figure 6**). However, GPC in areas north of 38.0° N was not related to the variation of latitude. The accumulation of sunlight hours during filling to maturity stage mainly improve GPC in these areas (**Figure 6**).

No more than 502.65 GDD during filling to maturity stage generated the lowest GPC across the study region (**Figure 4**). The negative relationship between latitude and GDD during filling to maturity stage together caused the decrease of GPC in areas south of 38.0° N (**Figure 4**). At least 515.88 GDD during filling to maturity stage was required to main high GPC in these areas (**Figure 4**). However, no such effect existed in areas north of 38.0° N, where 515.88 to 528.52 GDD during filling to maturity stage was related to lower GPC regardless of latitude variation (**Figure 4**).

**Figure 7:**
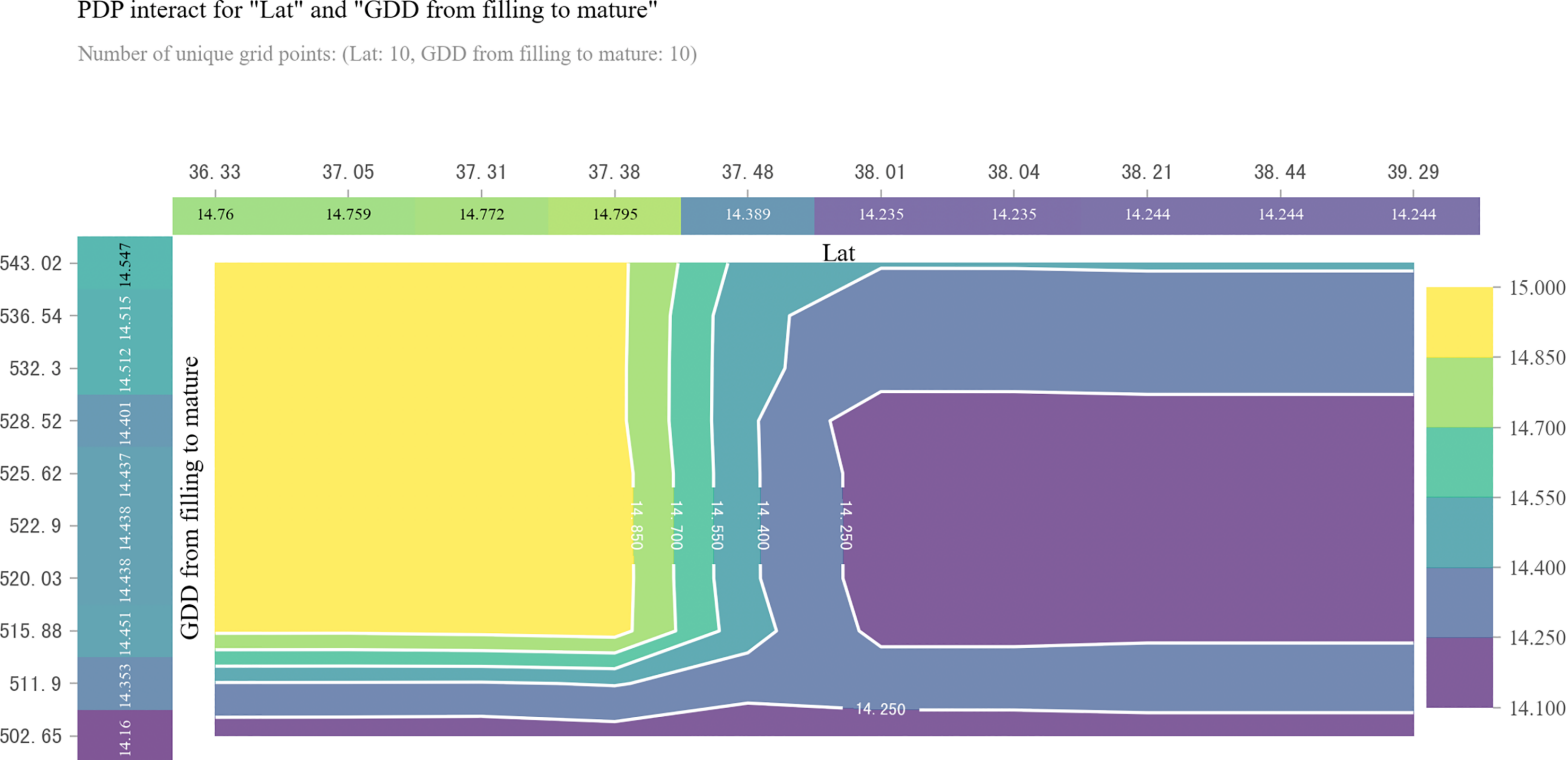
Two-dimensional partial dependence plot (2D PDP) for revealing the interaction effect of latitude and sunlight hours from sowing to maturity on grain protein content (GPC). The top horizontal label represents the variation in latitude (Lat). The left vertical label represents the variation in sunlight hours from sowing to maturity (SHavg). The right vertical label represents the variation in GPC.

When relative humidity during filling to maturity stage dropped to the range of 46.09% to 53.69%, it was related to higher GPC in areas farther south of 38.0° N (**Figure 7**). In areas north of 38.0° N, we observed the lowest GPC when relative humidity during filling to maturity stage varied from 55.02% to 58.99% (**Figure 7**). However, when relative humidity from filling to maturity was more than 58.99%, GPC increased with the increase of relative humidity from filling to maturity until it reached 66.76% (**Figure 7**).

## 4 Discussion

The observed mean winter wheat GPC of the Hebei Province was 14.5% ± 0.9% from 2006 to 2018, in line with its leading position in the production of high-protein wheat. The predicted GPC in this study suggested by the GBDT model was 14.4% ± 0.4%, similar to the observed mean value over those years. More importantly, our findings shed light on the key controlling factors of GPC after we examined different meteorological conditions during important phenological periods of winter wheat growth.

The effect of latitude on GPC as well as its major components (e.g., albumins, globulins, gliadins and glutenins) in wheat grain has been observed in previous work (Liu et al., 2016; Sun et al., 2018; Zahra et al., 2023). Our study confirmed the important effect of latitude on GPC at provincial scale. Generally, wheat GPC accumulates at higher latitudes on a large spatial scale. For example, wheat GPC presented a pattern of high in North China and low in South China (Liu et al., 2016). In line with this positive correlation, the conclusion drawn by Sun et al. (2018) showed improved GPC as the latitude increased from 32.6° N to 38.7° N across six main wheat production provinces in China. In contrast, another study suggested a declining trend of GPC when latitude increased from 32.1° N to 35.9° N within the Henan Province (Zhang et al., 2008). Both the positive and negative correlation between latitude and GPC was observed within our study region with latitude varying from 36.2° N to 40.0° N. GPC decreased with increasing latitude in areas south of 38.0° N (e.g., 36.2° N-38.0° N) and increased with increasing latitude in areas north of 38.0° N (e.g., 38.0° N-40.0° N). The inconsistent effect of latitude on GPC may be due to different responses of grain protein components to latitude. To be specific, gliadins and glutenins, the major storage protein factions, together constituted up to 80% of the total protein in wheat grain (Békés and Wrigley, 2016). The content of gliadins decreased with increasing latitude, while the content of glutenins increased (Zahra et al., 2023). If the increase rate of glutenins surpassed the decrease rate of gliadins, then GPC tended to increase with increasing latitude and vice versa. There is still great uncertainty about the change rate of gliadins and glutenins with latitude yet. This may be the cause of the inconsistent response of GPC to increasing latitude.

Our study underlined the spatial pattern and controlling meteorological factors of winter wheat GPC in Hebei Province were different in areas south and north of 38.0° N. GPC observed in areas south of 38.0° N was not subject to variations in sunlight hours. However, sunlight hours influenced GPC at higher latitudes. GPC in areas north of 38.0° N required more than 1500 hours of sunlight to improve (**Figure 6**). The identified positive correlation between GPC and latitude in high latitude regions was in fact indirectly caused by the significant positive relationship between latitude and sunlight hours. Sunlight represents light intensity that is vital for crop growth. Shading experiments to mimic reduced light intensity have demonstrated a reduction in leaf nitrogen concentration, depression of plant nitrogen uptake and nitrogen translocation to grain (Li et al., 2005; Mu et al., 2010; Shimoda and Sugikawa, 2020; Arenas-Corraliza et al., 2021). Our result highlighted abundant light for the whole growth season could contribute to GPC improvement.

GDD represents the thermal requirement for completing certain phenology (Tao et al., 2012). Our study showed that at least 515 GDD from filling to maturity contributed to the optimum GPC in the southernmost areas south of 38.0° N (**Figure 4**). Although GDD within the range of 515 to 530 corresponded with a slightly lower GPC in areas north of 38.0° N (**Figure 4**), the t-test showed insignificant difference (*p* = 0.76), suggesting that the interaction between GDD and latitude was mainly reflected in the region south of 38.0° N. Temperature is generally thought to greatly affect grain quality during the filling period (Zhang et al., 2019). Our study showed that temperature could impact final grain quality as early as during the jointing to flowering period. Temperature during jointing to flowering may influence crop processes related to nitrogen assimilated in vegetative organs and subsequently the ability to translocate nitrogen to the grain (Zhang et al., 1997; Fernie et al., 2022).

The importance of precipitation on GPC was previously highlighted in Mediterranean countries with rain-fed agriculture (Lee et al., 2013; Toscano et al., 2015). But our study suggested a limited influence of precipitation on GPC, given the fact that crops in Hebei Province were all well irrigated to maintain high yield. The annual averaged irrigation water volume was 2250 m^3^ per hectare during the recent five years. The influence of precipitation shortage may hard to be detected because irrigation can ensure enough water supply for crop growth. The influence of water excess and shortages on GPC would require examination over a controlled experiment where conditions are not ideal for productive agriculture. The high reliance of agriculture on irrigation in this region will lead to unfavorable risks. For example, the impact of climate change on water resources may pose threat to the sustainable development of agriculture in this region. Several studies found that high relative humidity tended to increase grain yield (Joshi et al., 2010; Mohammadi et al., 2020) and may consequently decrease GPC due to the growth dilution effect (Pleijel and Uddling, 2012). This assumption was partly supported by our results. We only observed lower GPC in areas south of 38.0° N when the relative humidity exceeded 53% within the acceptable relative humidity range for winter wheat growth. By contrast, another study debated that high relative humidity can increase wheat nitrogen assimilation and remobilization with dry mass accumulation at the same time, depending on soil fertility (Huang et al., 2011). Our result also confirmed this finding based on the following two points. First, we observed an improvement in GPC with moderately increased relative humidity from 59% to 67% in areas north of 38.0° N (**Figure 7**). Second, the farmland in Hebei Province is characterized by high soil available nitrogen (Pan et al., 2011). Our results highlighted the spatial relationship between GPC and meteorological variables was not linear at the provincial scale. This further indicates that identifying multiple piecewise linear relationships in nonlinear relationships may enhance the prediction of protein content by meteorological elements.

## 5 Conclusion

In conclusion, latitude played a prominent role in deciding regional GPC variation by forming two distinct variation patterns at the breakpoint of 38.0° N. The different variation patterns in areas south and north of 38.0° N were largely induced by the correlation between latitude and different meteorological factors. For Hebei Province, the high GPC in areas south of 38.0° N can be further enhanced by more than 515 GDD during filling to maturity period. Moreover, greater than 1500 sunlight hours for the whole growth season in Hebei Province helped to improve GPC in areas north of 38.0° N. In addition, relative humidity exceeding 59% was beneficial to GPC in areas north of 38.0° N.

## Data availability statement

The data that support the findings of this study are available on request from the corresponding author, Xinyue Zhang, upon reasonable request.

## Author contributions

All authors read and approved the final version of the manuscript. XZ conceived of the study. XZ designed the methodology and wrote the first draft of the manuscript. KC collected the data. KC and XZ conducted the analysis. KL reviewed and edited the manuscript. All authors contributed to the article and approved the submitted version.
